# Assessment of population-based input functions for Patlak imaging of whole body dynamic ^18^F-FDG PET

**DOI:** 10.1186/s40658-020-00330-x

**Published:** 2020-11-23

**Authors:** Mika Naganawa, Jean-Dominique Gallezot, Vijay Shah, Tim Mulnix, Colin Young, Mark Dias, Ming-Kai Chen, Anne M. Smith, Richard E. Carson

**Affiliations:** 1 PET Center, Department of Radiology and Biomedical Imaging, Yale University, New Haven, CT USA; 2 Molecular Imaging, Siemens Medical Solutions USA, Inc., Knoxville, TN USA

**Keywords:** ^18^F-FDG, Population-based input function, Whole body PET imaging, Patlak plot

## Abstract

**Background:**

Arterial blood sampling is the gold standard method to obtain the arterial input function (AIF) for quantification of whole body (WB) dynamic ^18^F-FDG PET imaging. However, this procedure is invasive and not typically available in clinical environments. As an alternative, we compared AIFs to population-based input functions (PBIFs) using two normalization methods: area under the curve (AUC) and extrapolated initial plasma concentration (*C*_P_*(0)). To scale the PBIFs, we tested two methods: (1) the AUC of the image-derived input function (IDIF) and (2) the estimated *C*_P_*(0). The aim of this study was to validate IDIF and PBIF for FDG oncological WB PET studies by comparing to the gold standard arterial blood sampling.

**Methods:**

The Feng ^18^F-FDG plasma concentration model was applied to estimate AIF parameters (*n* = 23). AIF normalization used either AUC(0–60 min) or *C*_P_*(0), estimated from an exponential fit. *C*_P_*(0) is also described as the ratio of the injected dose (*ID*) to initial distribution volume (*iDV*). *iDV* was modeled using the subject height and weight, with coefficients that were estimated in 23 subjects. In 12 oncological patients, we computed IDIF (from the aorta) and PBIFs with scaling by the AUC of the IDIF from 4 time windows (15–45, 30–60, 45–75, 60–90 min) (PBIF_AUC_) and estimated *C*_P_*(0) (PBIF_iDV_). The IDIF and PBIFs were compared with the gold standard AIF, using AUC values and Patlak *K*_i_ values.

**Results:**

The IDIF underestimated the AIF at early times and overestimated it at later times. Thus, based on the AUC and *K*_i_ comparison, 30–60 min was the most accurate time window for PBIF_AUC_; later time windows for scaling underestimated *K*_i_ (− 6 ± 8 to − 13 ± 9%). Correlations of AUC between AIF and IDIF, PBIF_AUC(30–60)_, and PBIF_iDV_ were 0.91, 0.94, and 0.90, respectively. The bias of *K*_i_ was − 9 ± 10%, − 1 ± 8%, and 3 ± 9%, respectively.

**Conclusions:**

Both PBIF scaling methods provided good mean performance with moderate variation. Improved performance can be obtained by refining IDIF methods and by evaluating PBIFs with test-retest data.

## Background

A whole body (WB) dynamic PET acquisition enables ^18^F-FDG parametric imaging. Full kinetic modeling analysis of ^18^F-FDG using WB dynamic PET requires tissue time-activity curves (TACs) measured by PET and the arterial input function (AIF). The Patlak plot model [1, 2] can then be applied to these data to compute the net influx parameter, *K*_i_, which is proportional to the glucose metabolic rate.

The AIF is obtained by collecting arterial blood samples and measuring the radioactivity concentration in the arterial plasma; these data are generally considered to be the gold standard. This invasive measurement can be associated with patient discomfort and additional exposure to personnel. Additionally, serial arterial blood sampling is not typically feasible in a clinical environment. Therefore, an alternative to arterial blood sampling for estimating the input function (IF) is desired for routine use. Several alternative methods have been proposed to replace the AIF: arterialized venous blood sampling [3], image-derived input function (IDIF) estimation [4–6], and population-based input function (PBIF) modeling [7–10]. Venous blood sampling is more convenient than arterial blood sampling, but it is still invasive, especially with arterialization, i.e., sampling blood from a hand immersed in 44 °C water [11]. Heating the hand causes a vascular dilatation and increases the blood flow to the hand, so that venous samples are similar to arterial samples [12].

Measures of blood activity can be obtained by WB PET scans that typically cover large arterial blood regions such as the left ventricle and aorta; however, the accuracy of IDIFs will be affected by body motion and partial volume effects. Furthermore, the injection must be performed with the patient on the bed in order to measure the early phase of the IDIF, further compromising a clinically established workflow. The PBIF method starts with the generation of a normalized average of measured arterial blood data from several subjects (template PBIF). The PBIF method assumes that the shape of the IFs of all subjects is the same. This assumption may be violated in some patients if tracer absorption differs. The PBIF method also requires the determination of an appropriate factor to scale the template PBIF for each patient, which is another possible source of error.

In this paper, we applied both IDIF and PBIF methods to ^18^F-FDG WB PET data of oncologic patients and compared the performance of these methods with the gold standard of arterial blood sampling denoted as AIF in this paper, by assessing the Patlak *K*_i_ values. To generate the template PBIF, we applied two normalization methods. These template PBIFs were normalized for each subject using several scaling factors: (1) a scaling factor consisting of injected dose (*ID*) and initial distribution volume (*iDV*) of ^18^F-FDG [10] and (2) the area under the curve (AUC) of the IDIF using several time windows. While there has been substantial literature over many years developing IDIFs and PBIFs, this paper has a number of unique characteristics: (1) use of a modern PET system to extract IDIF and assess tumor quantification, (2) comparison to gold standard arterial samples, (3) use of commercial algorithms to define the aorta region of interest (ROI), and (4) comprehensive evaluation of scaling methods for the PBIF.

## Material and methods

The abbreviations are listed in Table 1.

**Table 1:** Abbreviations

Abbreviations	Terms
AIF	Arterial input function
AUC	Area under the curve
COV	Coefficient of variation
ID	Injected dose
iDV	Initial distribution volume
IDIF	Image-derived input function
IF	Input function
PBIF	Population-based input function
PBIF_AUC_	Population-based input function created by normalizing AIFs by their AUC
PBIF_iDV_	Population-based input function created by normalizing AIFs by the initial ^18^F-FDG plasma concentration
ROI	Region of interest
sPBIF	Scaled population-based input function
sPBIF_AUC(t1–t2)_	Scaled population-based input function by the AUC of the IDIF in time window t1 min to t2 min
sPBIF_iDV_	Scaled population-based input function by the initial ^18^F-FDG plasma concentration
sPBIF_PLAS_	Scaled population-based input function by the average of the ratio of plasma samples to PBIF
TAC	Tissue time-activity curve
WB	Whole body

### Human subjects and PET scan procedure

A total of 35 subjects were recruited for this study (Table 2). All subjects provided written consent. The study was performed in accordance with the ethical standards as laid down in the 1964 Declaration of Helsinki and federal guidelines and regulations of the USA for the protection of human research subjects contained in Title 45 Part 46 of the Code of Federal Regulations (45 CFR 46).

**Table 2:** Demographics and injection parameters

Parameter	PBIF generation	PBIF validation
Number of subjects	23 (16M/7F)	12 (4M/8F)
Age (years)	41 ± 9	59 ± 15
Body height (m)	1.70 ± 0.09	1.71 ± 0.08
Body weight (kg)	91 ± 15	83 ± 14
BMI (kg m^−2^)	31.4 ± 5.4	28.1 ± 3.3
Injected dose (MBq)	252 ± 83	331 ± 30

The subjects were divided into 2 groups: a PBIF generation group (*n* = 23; 11 healthy controls (HCs) and 12 clinical subjects (post-traumatic stress disorder (*n* = 6), epilepsy (*n* = 3), cocaine addiction (*n* = 3))) and a PBIF validation group (*n* = 12; oncologic subjects). In the validation group, tumors or hypermetabolic nodes were located in palate, neck, thyroid, esophagus, axilla, lung, mediastinum, inguen, and femoral shaft.

^18^F-FDG was injected by pump using a 1-min infusion and arterial blood sampling was performed for 90 min in all subjects except for 1 subject (60 min). Discrete blood samples were manually drawn every 10 s from 0 to 90 s, every 15 s from 90 s to 3 min, and then at 4, 5, 6, 8, 10, 15, 20, 25, 30, 45, 60, 75, and 90 min post-injection. Samples were centrifuged to obtain plasma and then counted with a cross-calibrated well counter to produce the AIF in units of Bq/mL decay corrected to injection time.

PET scans were acquired for 90 min on a 4-ring Biograph mCT PET/CT scanner concurrently with arterial blood sampling for the PBIF validation group (*n* = 12). A single bed cardiac PET scan was acquired for the first 6 min, followed by continuous bed motion dynamic whole body scans (2 min × 4 passes, 5 min × 15 passes). The subjects were scanned from top of the head to the knee. The dynamic data were reconstructed using OSEM (2 iterations, 21 subsets) using point spread function recovery and time of flight information, with a matrix size of 400 × 400 and 5 mm full width at half maximum Gaussian post-reconstruction filtering. The data were corrected for attenuation, randoms, and scatter, but not for motion. The CT scan was not co-registered to PET since it was acquired immediately before the ^18^F-FDG injection. However the quality of the alignment was visually checked.

### Normalization of AIF

The first step to generate a template PBIF curve is to normalize the amplitude of each AIF. The AIFs from the PBIF generation group were normalized in two ways. The first method used the AUC from 0 to 60 min of the AIF. For the PBIF generation group, each AIF was divided by its AUC. The second method was to use the method proposed by Vriens et al. [10], denoted as the iDV (initial distribution volume) method. The AIFs were normalized with the extrapolated initial plasma concentration of ^18^F-FDG (*C*_P_*(0)). *C*_P_*(0) is the expected plasma concentration under the assumption of instantaneous mixing of ^18^F-FDG at *t* = 0 [13]. *C*_P_*(0) was obtained by fitting a portion of the curve (5 ≤ *t* ≤ 30 min) with an exponential function (*C*_P_*(*t*) = *C*_P_*(0)exp(-*αt*)) [14]. Each AIF was divided by its estimated *C*_p_*(0).

The *iDV* is the ratio of the injected dose (*ID*) to the initial FDG concentration, *C*_P_*(0 )[14] and is effectively the volume of blood that accounts for the early distribution of tracer throughout the body. The value of *iDV* can be approximated noninvasively using the subject body weight and height as follows:

1 $$ iDV\;\left[L\right]=c{\left(\mathrm{height}\left[\mathrm{m}\right]\right)}^h\kern0.5em {\left(\mathrm{weight}\left[\mathrm{kg}\right]\right)}^w $$

where *c*, *h*, and *w* are pre-determined coefficients. These three coefficients were estimated from the individual values of *iDV* (=*ID*/*C*_P_*(0)), height, and weight of the subjects in the PBIF generation group. Specifically the coefficients *h* and *w* were first determined by minimizing the coefficient of variation of *c* (COV_c_) [8, 10]. Then, the coefficient, *c*, was determined as the mean of *iDV*/[(height)^*h*^(weight)^*w*^] among subjects.

### Creation of PBIF

In the next step to generate a template PBIF curve, the normalized AIF (by AUC and iDV methods) was modeled using a compartment model that describes tracer behavior in the circulatory system proposed by Feng et al. [9].

2 $$ {C}_P(t)=\left\{\begin{array}{c}0\;\mathrm{if}\kern0.5em t<\tau \\ {}\left[{A}_1\left(t-\tau \right)-{A}_2-{A}_3\right]{e}^{-{\lambda}_1\left(t-\tau \right)}+{A}_2{e}^{-{\lambda}_2\left(t-\tau \right)}+{A}_3{e}^{-{\lambda}_3\left(t-\tau \right)}\kern0.1em \mathrm{if}\;t\ge \tau \end{array}\right. $$

where *λ*_1_, *λ*_2_, and *λ*_3_ are the eigenvalues of the model; *A*_1_, *A*_2_, and *A*_3_ are the coefficients; and *τ* is the delay constant.

Since Feng’s model describes the plasma as an impulse response function, i.e., from a true bolus injection, the model was convolved with a rectangular function (*f*(*t*) = 1, 0 ≤ *t* ≤ 1; *f*(*t*) =0, otherwise) to take into account our injection protocol (1-min bolus). Feng’s model was applied twice. First, nonlinear least square fitting was applied to obtain the 7 parameters for each subject of the PBIF generation group. Each model-fitted normalized AIF was corrected for its estimated delay (*τ*) and then averaged. Next, Feng’s model was again applied to the average curve to obtain a final parameter set. The fitted PBIFs using both normalization methods are thereafter denoted as PBIF_AUC_ and PBIF_iDV_. In the PBIF generation group, the shapes of two PBIFs were compared as follows. First, the parameters (*λ*_1_, *λ*_2_, *λ*_3_) and the ratios of scale parameters (*A*_2_/*A*_1_, *A*_3_/*A*_1_) were compared between PBIF_AUC_ and PBIF_iDV_. Next, the Patlak *K*_i_ values were compared using PBIF_AUC_ and PBIF_iDV_ that were scaled to have the same AUC.

### IDIF

In the validation group, an IDIF was generated from descending aorta region automatically defined on the CT, which was used for PET attenuation correction, by a cylindrical ROI using the vendor’s ALPHA technology. The organ region of interest prediction was conducted using a learning-based algorithm [15] for automatic medical image annotation. Multiple focal anatomical structures were detected by a learning-by-example landmark detection algorithm and then inconsistent findings were eliminated through a robust sparse spatial configuration algorithm.

### Subject scaling of PBIF for validation

The template PBIFs must be scaled for each individual subject, and the scaled PBIF is denoted as sPBIF. For PBIF_AUC_, the scaling factor was determined based on the tail part of IDIF (from 15 to 90 min post-injection) using 4 different time windows. The length of the time window for scaling was 30 min, i.e., the same as the length for Patlak plot computation (see below). Multiple time windows were used as it was likely that effects such as motion and partial volume effects would produce differences in bias. Four different time windows (15–45, 30–60, 45–75, and 60–90 min) were used to scale the template PBIFs by multiplication by the AUC of the IDIF in each window (sPBIF_AUC(15–45)_, sPBIF_AUC(30–60)_, sPBIF_AUC(45–75)_, sPBIF_AUC(60–90)_). For PBIF_iDV_, the scaling factor was computed using the injected dose and the estimated *iDV* using each subject’s weight and height with Eq. 1. To evaluate the robustness of *iDV* estimates, *iDV* was estimated in 3 ways, using the coefficients *c*, *w*, and *h* from this study, and also with the coefficients from 2 previous studies [8, 10]. In addition, to evaluate the results that could be obtained with the “best possible” scaling factor (i.e., using the subject’s plasma data), we also computed the ratio of the measured plasma to PBIF_iDV_ at 4 time points (30, 45, 60, and 75 min post-injection) for each subject. The average of these 4 ratios was used as a scaling factor to obtain sPBIF_PLAS_.

In total, 9 estimated IFs (1 IDIF, 3 sPBIF_iDV_, 1 sPBIF_PLAS_, and 4 sPBIF_AUC_) were obtained per scan for validation.

### Comparison of the scaled PBIFs with IDIF and AIF

The performance of the 9 estimated IFs was compared in the validation group using the AIF as the gold standard. Two outcome measures were used to evaluate the performance: the AUC of the IF and the Patlak *K*_i_. ROIs for tumors or hypermetabolic nodes were manually delineated on multiple slices of the summed (60–90 min post-injection) PET images. The size of ROI was 3.46 ± 2.21 mL (one ROI per subject). The ROIs were applied to generate time-activity curves (TACs). The net influx rate constant (*K*_i_) and the exchangeable distribution volume (*V*_e_, intercept of Patlak plot) were determined for the ROI TACs using each IF and Patlak analysis applied to the period of 60–90 min post-injection. Specifically, we used a multilinear analysis to estimate *K*_i_ and *V*_e_ using the following equation:

3 $$ C(t)={K}_{\mathrm{i}}{\int}_0^t{C}_{\mathrm{P}}\left(\tau \right)\; d\tau +{V}_{\mathrm{e}}{C}_{\mathrm{P}}(t),t>{t}^{\ast } $$

### Effect of whole blood to plasma ratio

The PBIF curves generated here were created from plasma data. However, in the above assessment, the IDIF, which measures whole blood, was not corrected for the whole blood to plasma ratio, and PBIF_AUC_ was scaled using the AUC of the uncorrected IDIF. In a separate analysis, we assessed the effect of the difference between concentrations of ^18^F-FDG in whole blood and plasma by determining the resulting bias in *K*_i_. The whole blood to plasma ratio was computed from 40 s to 90 min post-injection in the PBIF validation group.

### Statistical analysis

Correlations between the AUC and *K*_i_ with the estimated IFs and the AIF were assessed by Pearson *r*, mean bias, and standard deviation (SD) of bias. Statistical analysis was performed by Prism 8 (GraphPad Software). All kinetic modeling was performed with in-house programs written with IDL 8.0 (ITT Visual Information Solutions, Boulder, CO).

## Results

### Creation of PBIF

The parameters from fitting of the AIFs by Feng’s model using the AUC and *C*_P_*(0) (*ID*/*iDV*) normalizations are summarized in Table 3. The shape-related parameters (*λ*_1_, *λ*_2_, *λ*_3_) were very similar between PBIF_AUC_ and PBIF_iDV._ The values of the relative amplitudes *A*_2_/*A*_1_ and *A*_3_/*A*_1_ were similar between the two PBIFs: 0.010 and 0.008 for PBIF_AUC_ and 0.009 and 0.006 for PBIF_iDV_, respectively.

**Table 3:** Parameters of template PBIFs

Parameter	PBIF_AUC_	PBIF_iDV_
*τ* (min)	0.587	0.613
*A*_1_^a^	88.9	3.1
*λ*_1_ (min^−1^)	6.66	7.42
*A*_2_^a^	0.91	0.027
*λ*_2_ (min^−1^)	0.21	0.22
*A*_3_^a^	0.68	0.020
*λ*_3_ (min^−1^)	0.012	0.012

The two PBIF columns reflect different normalization methods applied to the PBIF generation group (see text for details)

^a^Units for the amplitude (*A*) values is [/min] for PBIF_AUC_ and [unitless] for PBIF_iDV_

To compare the two PBIFs, tests were performed with the two PBIFs scaled to have the same AUC. In that case, Patlak *K*_i_ values using PBIF_AUC_ were almost identical to those using PBIF_iDV_ (*K*_i_(PBIF_AUC_) = 0.994 × *K*_i_(PBIF_iDV_) − 0.002, *R*^2^ = 1.000), indicating that there is no meaningful difference between the shapes of the two PBIFs.

Comparing the contribution of the terms of Eq. 2 to the PBIF, the third term (${A}_3{e}^{-{\lambda}_3\left(t-\tau \right)}$) accounted for > 95% of the PBIF after 16 min post-injection.

### IDIF

**Fig. 1:**
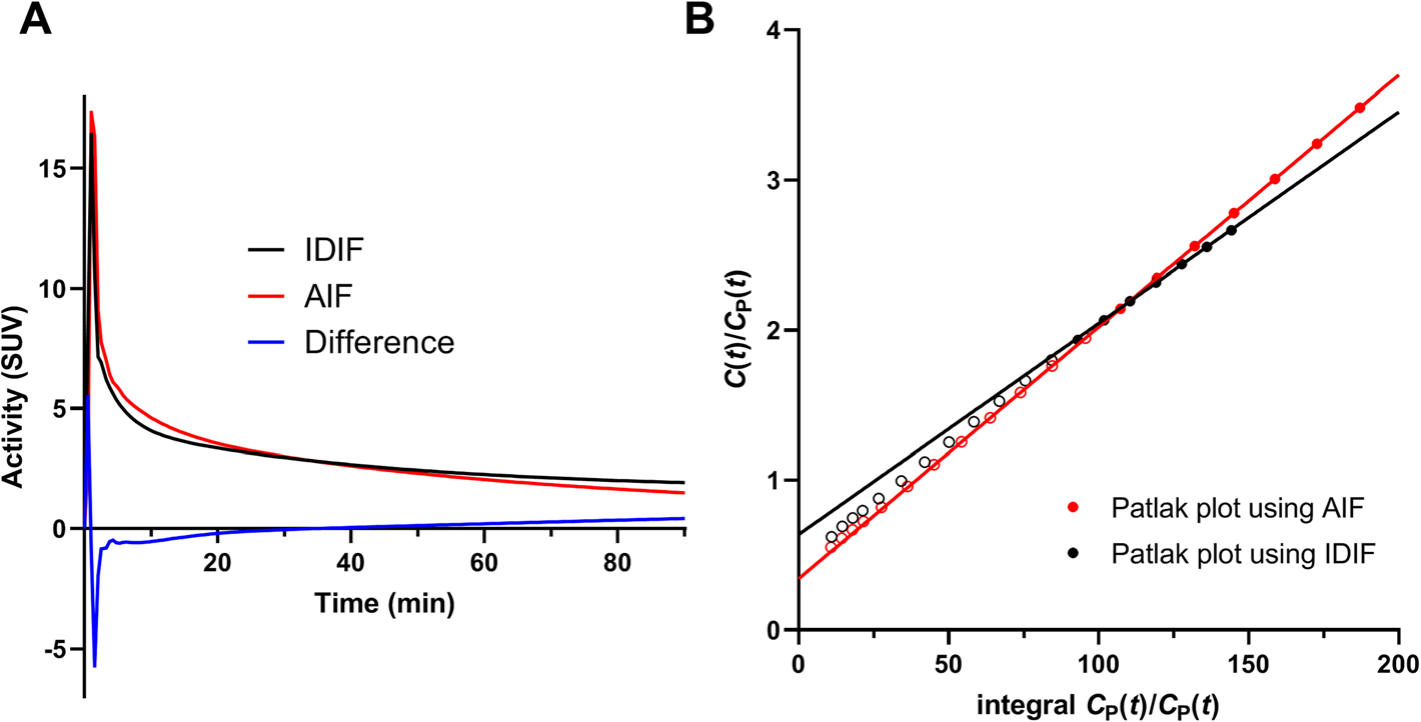
**a** Typical example of IDIF (black curve), AIF (red), and difference (IDIF − AIF; blue). **b** Patlak plots using IDIF (black) and AIF (red); solid lines show the portion of the plot used to estimate *K*_i_. In this case, the bias of AUC was 0.3% and the bias of *K*_i_ was − 16%

In the validation group, the volume of the aorta ROI was 1.55 ± 0.11 mL. Figure 1a shows a comparison of a typical IDIF and its corresponding AIF. The IDIF tends to undershoot the AIF at early times (*t* < 20 min) and overshoot it at late times (*t* > 30 min), with varying degree of under/overshoot among subjects (%difference, − 7% ± 8% (*t* < 20 min) and 13% ± 12% (*t* > 30 min)). Fitting Feng’s model to IDIFs and AIFs, the third eigenvalue *λ*_3_ of the IDIF was significantly smaller than that of AIF (IDIF, 0.008 ± 0.002 min^−1^and AIF, 0.011 ± 0.002 min^−1^, *P* = 0.008).

### Subject scaling of PBIF for validation

The median *iDV* was 13.1 L (mean ± SD = 13.0 ± 1.7), which corresponds to 0.14 L/kg body weight. Table 4 shows the three estimated coefficients (*c*, *h*, *w*) in our study (from the PBIF generation group) compared to previous references. Those coefficients were used to predict *C*_P_*(0) and compare to the actual values from blood samples in the validation group. Using values in this study, differences were acceptable (3 ± 8%). For the literature values, although the coefficients themselves were quite different, the percent bias of the estimated *C*_P_*(0) was reasonable, especially for the values from Vriens et al. [10].

**Table 4:** Comparisons of coefficients and *C*_P_*(0)

Reference	Coefficients			*C*_P_*(0)	
	*c*	*h*	*w*	%Bias	%SD
Shiozaki et al. [8]	1.55	0.80	0.35	16%	10%
Vriens et al. [10]	0.533	1.257	0.582	− 7%	6%
This study	1.18	0.68	0.45	3%	8%

Bias and SD of *C*_P_*(0) were estimated using the PBIF generation group (*n* = 23)

### Comparison of the scaled PBIFs with IDIF and AIF

In the validation group, comparisons between AUC(0–90 min) and Patlak *K*_i_ with respect to the AIF values are shown in Tables 5 and 6, respectively.

**Table 5:** Comparison of AUC(0–90 min) between the estimated IFs (*n* = 12)

	IDIF	sPBIF_AUC_				sPBIF_iDV_			sPBIF_PLAS_
		15–45 min	30–60 min	45–75 min	60–90 min	Shiozaki et al. [8]	Vriens et al. [10]	This study	
*R*^2^	0.91	0.93	0.94	0.93	0.88	0.86	0.88	0.90	0.93
Bias	1%	− 1%	3%	9%	19%	11%	− 8%	− 1%	1%
SD	5%	6%	6%	7%	10%	6%	8%	5%	5%

**Table 6:** Comparison of *K*_i_ between the estimated IFs

Number of subjects		IDIF	sPBIF_AUC_				sPBIF_iDV_			sPBIF_PLAS_
			15–45 min	30–60 min	45–75 min	60–90 min	Shiozaki et al. [8]	Vriens et al. [10]	This study	
*n* = 10^a^	*R*^2^	0.99	0.98	0.98	0.98	0.99	0.97	0.96	0.97	0.99
	Bias	− 9%	3%	− 1%	− 6%	− 14%	− 8%	12%	3%	2%
	SD	10%	8%	8%	8%	9%	9%	11%	9%	6%
*n* = 12	*R*^2^	0.99	0.99	0.99	0.99	0.99	0.98	0.97	0.97	0.99
	Bias	− 16%	4%	0%	− 6%	− 13%	− 9%	11%	2%	3%
	SD	20%	9%	9%	8%	9%	11%	13%	12%	6%

^a^Two scans with the lowest *K*_i_ values were removed

**Fig. 2:**
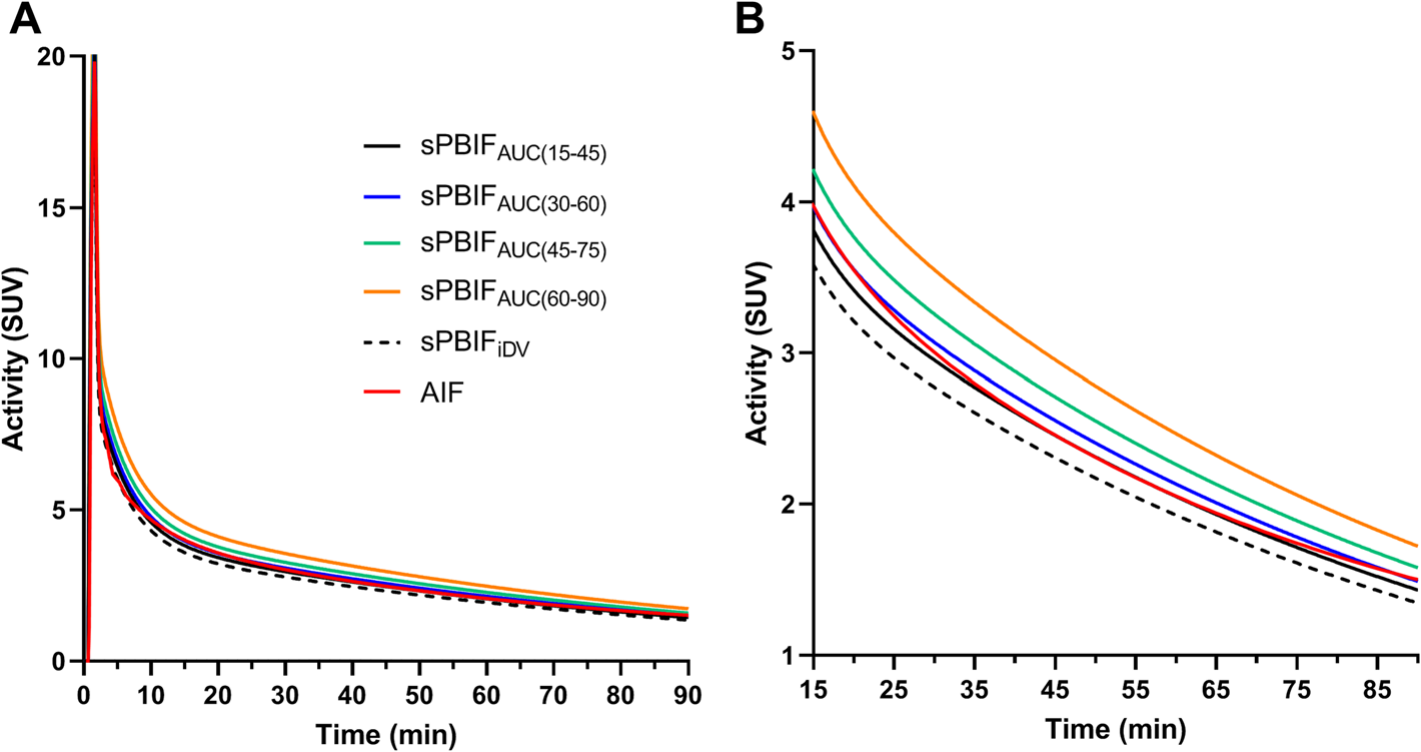
Typical example of sPBIFs and AIF **a** for the full 90 min and **b** from 15 min to 90 min post-injection. These data are from the same subject used in Fig. 1

For AUC, the early time windows, 15–45 min or 30–60 min, for scaling PBIF_AUC_ provided similarly good performance (0–90 min) in terms of Pearson *r*, bias, and SD (Table 5). Later time windows produced poorer correlation and overestimated the AUC(0–90 min). Typical sPBIFs are shown in Fig. 2 where the differences in scaling are best visualized in the tail of the curve. The correlation, bias, and SD were similar between IDIF, sPBIF_AUC_ with the best time window, sPBIF_iDV_, and sPBIF_PLAS_ (correlation, 0.90–0.94; bias, − 1 to 3%; SD, 5–6%).

**Fig. 3:**
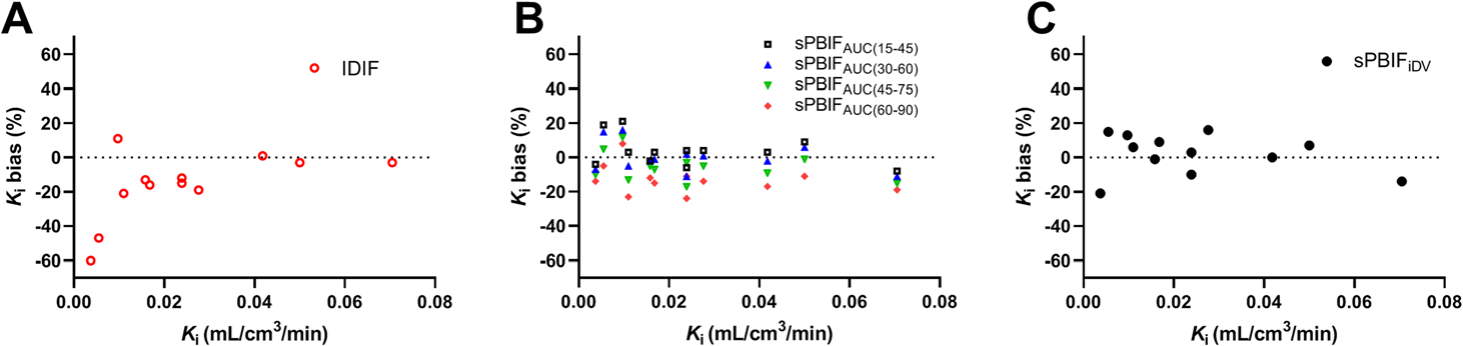
Individual values of *K*_i_ bias using different input functions compared to *K*_i_ estimated with the AIF. **a** IDIF. **b** sPBIF_AUC_. **c** sPBIF_iDV_. Each symbol represents the *K*_i_ derived from the tumor TAC of each subject

Figure 3 shows individual *K*_i_ bias values using the IDIF or any of the sPBIFs, with *K*_i_ estimated using the AIF as the gold standard. The %bias was particularly large (− 47 and − 60%; Fig. 3a) for small *K*_i_ values (< 0.01 mL/min/cm^3^) with the IDIF. Therefore, the *K*_i_ bias (Table 6) was calculated in two ways, i.e., with and without these two tumors. Unlike the IDIF method, the *K*_i_ bias using all PBIF values was not affected by the magnitude of *K*_i_ (Fig. 3b, c).

When AUC was overestimated, *K*_i_ was generally underestimated (Table 6). Patlak *K*_i_ determined by the IDIF was lower than the gold standard values (using the AIF) (− 9%), although the correlation was similar to those of other PBIFs (0.99–1.00). For sPBIF_AUC_, *K*_i_ was underestimated when using late time windows to scale the PBIF_AUC_ (− 14% using 60–90 min). Conversely, using early time windows for scaling, the correlation, bias, and SD of sPBIF_AUC_ was closest to those of sPBIF_PLAS_, which represents the best-possible outcome. For sPBIF_iDV_, using scaling coefficients from this study, the mean bias was low, the SD of the bias was similar to other methods, and the correlation lower than with sPBIF_AUC_. Using scaling coefficients from other published studies for sPBIF_iDV_ led to larger mean bias and similar correlation and SD.

### Effect of whole blood to plasma ratio

**Fig. 4:**
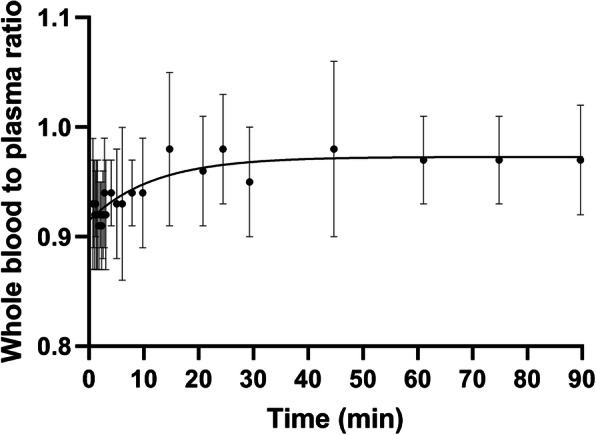
Mean and SD of whole blood to plasma ratio in PBIF validation group with the fitted curve. The mean values were fitted to a one phase decay model (ratio = − 0.06 exp(− 0.085 × time) + 0.97)

The whole blood to plasma ratio increased from a mean of 0.93 to 0.97 over 90 min (Fig. 4): The whole blood/plasma curve could be described by the function 0.97 − 0.06 × exp(− 0.08 × *t*). The mean ratio did not differ between 30 min (0.95 ± 0.05) and at 90 min post-injection (0.97 ± 0.05). The mean whole blood to plasma ratio was 0.97 ± 0.04 (15–45 min), 0.96 ± 0.03 (30–60 min), 0.97 ± 0.03 (45–75 min), 0.97 ± 0.04 (60–90 min), and 0.94 ± 0.03 (40 s–90 min). Applying the above mean whole blood to plasma ratio values for correction to the IDIF increased its value, so *K*_i_ values became even more underestimated: the mean bias of *K*_i_ became − 14% (IDIF), 0% (sPBIF_AUC(15–45)_), − 4% (sPBIF_AUC(30–60)_), − 9% (sPBIF_AUC(45–75)_), and − 16% (sPBIF_AUC(60–90)_) instead of the values in Table 6 (*n* = 10).

## Discussion

This study compared the performance of PBIFs with different normalization and scaling methods for the purpose of measuring the Patlak uptake constant *K*_i_ for ^18^F-FDG. The PBIFs were compared to IDIF and AIFs, with the latter used as the gold standard.

Two forms of the PBIF were generated from arterial sample data using two normalization methods (AUC or *C*_P_*(0)) and were first compared. The *K*_i_ values using PBIF_AUC_ were almost identical to those using PBIF_iDV_. This suggests that the PBIF shape was not affected by the different normalization methods. Therefore, the comparison among PBIFs was reduced to the comparison of scaling factors.

To apply the PBIFs without the need for blood sampling, we tested two scaling methods. We also scaled the PBIF using the measured plasma samples for each scan to define the best achievable results by PBIF. Four plasma samples at 30, 45, 60, and 75 min post-injection were used for scaling to reduce effects of measurement noise in the plasma. The sPBIF_PLAS_ overestimated *K*_i_ by 2 ± 6 %, due to slight differences in IF shape between subjects. Thus, ideally, a blood-free PBIF method could achieve comparable results.

One scaling method used a part of the IDIF. In WB PET imaging, large blood pools are always available. As shown in Fig. 1, the estimated IDIF showed a consistent pattern compared to the AIF, with undershoot at early times and overshoot at late times, perhaps due to partial volume averaging, but the magnitude of under/overshoot was different among subjects. Therefore, the Patlak *K*_i_ was significantly underestimated using the PBIFs scaled by the late AUC values from the IDIF. The best time window for scaling (in terms of minimum bias) was 30–60 min (bias, − 1% and SD, 8%; Table 6). In that case, however, the required scan time would be 1 h, 30–60 min to measure the part of the IDIF used for scaling, and 60–90 min for Patlak *K*_i_. Note that the SD of bias was very similar for all sPBIF_AUC_ time periods; thus, if a mean bias was acceptable, e.g., if that bias was consistent across scans in the same patient, then later time periods could be used for scaling, providing a short scan.

The second scaling method used the estimated *C*_p_*(0), the extrapolated initial ^18^F-FDG plasma concentration. This scaling approach has potential advantages since it does not require the IDIF for scaling and thus has a short scan and is not subject to effects of body motion and partial volume effect on the IDIF. Vriens et al. [10] reported a median *iDV* of 0.168 L/kg, slightly higher than the value in our study (0.144 L/kg). We fitted the *iDV* equation (Eq. 1) using the same method as Shiozaki et al. and Vriens et al. and found quite different values for the estimated coefficients (*c*, *h*, *w*). The estimated *C*_P_*(0) values using the injected dose and these coefficients were compared with the extrapolated *C*_P_*(0) values measured from the AIF. Not surprisingly, the bias of *C*_P_*(0) was smallest using our fitted parameters. The coefficient estimation might be affected by the study population or other methodological details. For example, the difference in body habitus of the study subjects at different sites might affect the results. Also, the estimation is affected by the correlation between height and weight which introduces instability in the parameters *h* and *w*. Patlak *K*_i_ estimated with this PBIF scaling method produced minimal bias and similar SD to the other scaling methods.

The mean biases of AUC(0–90 min) using IDIF, sPBIF_AUC_ with early time windows, and sPBIF_iDV_ were all minimal. However, a large negative mean bias of *K*_i_ with the IDIF was found, which was much larger than the other PBIF methods. Specifically, *K*_i_ with the IDIF was greatly underestimated (as a percentage) for small *K*_i_ values, while this was not observed for *K*_i_ with PBIF (Fig. 3). This difference in the *K*_i_ bias is due to the differences in the shapes of the IDIF and the AIF. The input function parameter *λ*_3_ (the terminal clearance rate) of the IDIF was much smaller than that of the AIF or the PBIFs, i.e., the IDIF showed slower clearance than the other IFs, resulting in large % underestimation of *K*_i_ for small *K*_i_ values.

To clarify this finding, we performed a simulation to assess the effect of *λ*_3_ on *K*_i_ estimates for large and small *K*_i_ values. Three IFs were computed using different *λ*_3_ values (0.012, 0.0084, 0.0048 min^−1^) (Figure S1-A) with all normalized to have the same AUC. Two TACs were computed using the input function with *λ*_3_ = 0.012 (Figure S1-B) having different *K*_i_ values (0.0077, 0.077 mL/min/cm^3^) but the same *V*_e_ (0.42). The Patlak plot was computed for these two TACs using three IFs, i.e., the correct IF and the two with slower terminal clearance (Figure S1-C and D). As shown in Table S1, *K*_i_ was underestimated, with much larger percent bias for small *K*_i_ values using the IFs with small *λ*_3_ values. The underestimated *K*_i_ was compensated by an overestimated intercept value, which has a larger error for larger *K*_i_.

In several past reports [10, 16], the IDIF, which measures whole blood, was used as IF without correction for the difference between concentrations of ^18^F-FDG in whole blood and plasma, assuming these differences are small [17]. In our study, we also used the uncorrected IDIF for Patlak analysis (Table 6). To assess this effect, the whole blood to plasma ratio was computed. Mean whole blood to plasma ratio increased monotonically from 0.93 to 0.97 over 90 min (i.e., the mean plasma to whole blood ratio decreased from 1.09 to 1.03). Similar results were reported previously (1.09 to 1.04 [11] and 1.12 to 1.07 [18] over 90 min). When the whole blood to plasma ratio is taken into consideration, mean underestimation of *K*_i_ by the IDIF method worsened slightly.

Several ^18^F-FDG tumor imaging guidelines reviewed in [19] suggested that a static scan should start at 30~40 min or 50~70 min post-injection, but an ideal time window (length and starting time) for tumor Patlak analysis is not clearly defined. In a brain study using healthy subjects, Lucignani et al. [20] reported that Patlak *K*_i_ is stable using a 30-min window in the interval between 45 and 120 min post-injection. In our study, we used a 60–90-min time window for Patlak analysis; this time period can also be used to generate a static SUV image by appropriate image averaging.

Comparing the results of our scaled PBIF methods, sPBIF_AUC(30–60)_ and sPBIF_iDV_ produced similarly small bias and high correlation coefficients in Patlak *K*_i_ estimation. In the PBIF_AUC_ method, no bias will be introduced due to an inaccurate dose calibrator cross-calibration to the PET scanner; however, errors in this calibration affect the PBIF_iDV_ method. PBIF_AUC(30–60)_ requires a 1-h scan when the Patlak time window is set from 60 to 90 min, while the PBIF_iDV_ requires scan time for the Patlak analysis only. Also, measurement of body weight, height, and injected dose is simpler than obtaining IDIF curves, depending on the available tools in each clinical environment. Therefore, PBIF_iDV_ would provide a simple protocol than PBIF_AUC(30–60)_. Using the methodology shown here, both approaches showed acceptable performance. sPBIF_AUC_ has slightly better performance, but sPBIF_iDV_ should be easier to implement in clinical setting, although some site-specific tuning of the iDV coefficients may be necessary.

In addition to considering mean bias, the SD of bias (~ 9%) for all sPBIF methods was larger than the best possible attainable value using the subject’s own plasma data (sPBIF_PLAS_, 6%). Since variances add in quadrature, this difference in SD suggests that an additional error of 6–7% is introduced by the IDIF AUC and iDV scaling methods. While it is not clear how to improve the iDV scaling method, IDIF performance would likely be improved by changing the shape of the ROI, as well as applying motion correction and partial volume correction. Since the IDIF ROI was defined from the CT, we assessed the effects of misalignment between the CT and PET on the AUC of the IDIF. The IDIF ROI was shifted by 1 to 6 voxels (i.e., 2 to 12 mm) in the *x* (left-right), *y* (anterior-posterior), and *z* (superior-inferior) directions, and we determined the maximum misalignment in each direction leading to ≤ 5% decrease in the AUC (15–45, 30–60, 45–75, and 60–90 min) from the shifted ROI. The most sensitive directions to misalignment were *y* (5 to 7 mm) and *x* (6 to 11 mm); the *z* direction showed minimal effects, as expected. The earlier time window was more sensitive to misalignment due to the higher contrast between the aorta and background. Partial volume effects would be a major contributing factor to the overestimation of AUC, especially in later time windows, as seen in Table 5 (19% overestimation of AUC(0–90 min) using sPBIF_AUC(60–90)_). If the quality of the IDIF ROI is improved, e.g., with motion and partial volume corrections, so that the later part of the IDIF can provide an accurate value, then the bias of *K*_i_ using PBIF_AUC(60–90)_ would be improved. In particular, in a typical clinical protocol, where the PET scan begins at 60 min, there will be less delay between CT and PET scans, so motion issues would likely be reduced. Also, we believe that using the imaging data to directly quantify the IF is of value, since day-to-day variation in the IF cannot be captured by the iDV method.

As described above, we assessed relative performance of the methods by calculating accuracy (mean % bias) and variability (SD of % bias). Both of these measures are relevant, although the relative importance depends on the clinical question. A small mean bias compared to the AIF means that the method is intrinsically accurate over the entire patient group. However, the SD of the bias across subjects and tumors should also be considered. If the SD is large, then the ability to reliably measure changes in tracer uptake between scans of the same patient may be poor. Alternatively, if large SD across patients is caused by subject-specific biases, e.g., due to IDIF ROI definition (excluding motion effects), which remain consistent across scans, then such variability may be clinically acceptable if the goal is to assess treatment response. Thus, the best way to fully assess the performance of PBIFs would be with test-retest data using the reproducibility of the estimated *K*_i_ as the key outcome measure.

Recent improved detector technology and clinical application demands led to the development of total body PET systems [21, 22], such as the uEXPLORER [23, 24] and PennPET Explorer [25]. Access for arterial blood sampling site is challenging in these systems. However, since the aorta is always in the field of view and the acquired dynamic data will have lower noise, the PBIF methods will be useful and compatible with these total body PET scan systems.

## Conclusions

In this paper, using a modern PET system, we assessed and optimized IDIFs and PBIFs using arterial blood samples and commercial software to define the IDIF ROI. We applied these IDIF and PBIF methods for FDG oncological WB PET studies. The PBIF methods scaled by either IDIF AUC or *ID* and *iDV* showed good performance, with a small mean bias and moderate variability, whereas the IDIF method produced negative mean bias of *K*_i_. Further improvements in accuracy and precision can be obtained with motion correction and partial volume corrections.

## Supplementary information

**Additional file 1: Figure S1.** (**A**) Three input functions simulated with different *λ*_3_ values (0%, 30%, 60% lower than mean value of 0.012 min^-1^) and the same area under the curve. Dotted curves show the difference from the input function with *λ*_3_=0.012; (**B**) two time-activity curves (TACs) computed using the input function (*λ*_3_=0.012). These curves have different *K*_i_ and the same *V*_e_ values, as specified in the legend; (**C**) Patlak plots of the TAC with the low *K*_i_ using the three input functions; (**D**) Patlak plots of the TAC with the high *K*_i_ using three input functions. Note the difference in *y*-axis scaling of (**C**) and (**D**). **Table S1.** Effect of *λ*_3_ of input function on the *K*_i_ estimation

## Data Availability

The datasets used and/or analyzed during the current study are available from the corresponding author on reasonable request.

## References

[1] Patlak CS, Blasberg RG, Fenstermacher JD (1983). Graphical evaluation of blood-to-brain transfer constants from multiple-time uptake data. J Cereb Blood Flow Metab.

[2] Karakatsanis NA, Zhou Y, Lodge MA, Casey ME, Wahl RL, Zaidi H (2015). Generalized whole-body Patlak parametric imaging for enhanced quantification in clinical PET. Phys Med Biol.

[3] van der Weerdt AP, Klein LJ, Visser CA, Visser FC, Lammertsma AA (2002). Use of arterialised venous instead of arterial blood for measurement of myocardial glucose metabolism during euglycaemic-hyperinsulinaemic clamping. Eur J Nucl Med Mol Imaging.

[4] Chen K, Bandy D, Reiman E, Huang SC, Lawson M, Feng D (1998). Noninvasive quantification of the cerebral metabolic rate for glucose using positron emission tomography, 18F-fluoro-2-deoxyglucose, the Patlak method, and an image-derived input function. J Cereb Blood Flow Metab.

[5] Asselin MC, Cunningham VJ, Amano S, Gunn RN, Nahmias C (2004). Parametrically defined cerebral blood vessels as non-invasive blood input functions for brain PET studies. Phys Med Biol.

[6] van der Weerdt AP, Klein LJ, Boellaard R, Visser CA, Visser FC, Lammertsma AA (2001). Image-derived input functions for determination of MRGlu in cardiac (18)F-FDG PET scans. J Nucl Med.

[7] Takikawa S, Dhawan V, Spetsieris P, Robeson W, Chaly T, Dahl R (1993). Noninvasive quantitative fluorodeoxyglucose PET studies with an estimated input function derived from a population-based arterial blood curve. Radiology..

[8] Shiozaki T, Sadato N, Senda M, Ishii K, Tsuchida T, Yonekura Y (2000). Noninvasive estimation of FDG input function for quantification of cerebral metabolic rate of glucose: optimization and multicenter evaluation. J Nucl Med.

[9] Feng D, Huang SC, Wang X (1993). Models for computer simulation studies of input functions for tracer kinetic modeling with positron emission tomography. Int J Biomed Comput.

[10] Vriens D, de Geus-Oei LF, Oyen WJ, Visser EP (2009). A curve-fitting approach to estimate the arterial plasma input function for the assessment of glucose metabolic rate and response to treatment. J Nucl Med.

[11] Phelps ME, Huang SC, Hoffman EJ, Selin C, Sokoloff L, Kuhl DE (1979). Tomographic measurement of local cerebral glucose metabolic rate in humans with (F-18)2-fluoro-2-deoxy-D-glucose: validation of method. Ann Neurol.

[12] Goldschmidt SI, Light AB (1925). Method of obtaining from veins blood similar to arterial blood in gaseous content. J Biol Chem.

[13] Sadato N, Tsuchida T, Nakaumra S, Waki A, Uematsu H, Takahashi N (1998). Non-invasive estimation of the net influx constant using the standardized uptake value for quantification of FDG uptake of tumours. Eur J Nucl Med.

[14] Goodman LS, Gilman A, Brunton LL, Hilal-Dandan R, Knollmann BC (2018). Goodman & Gilman's the pharmacological basis of therapeutics.

[15] Tao Y, Peng Z, Krishnan A, Zhou XS (2011). Robust learning-based parsing and annotation of medical radiographs. IEEE Trans Med Imaging.

[16] Zanotti-Fregonara P, Maroy R, Comtat C, Jan S, Gaura V, Bar-Hen A (2009). Comparison of 3 methods of automated internal carotid segmentation in human brain PET studies: application to the estimation of arterial input function. J Nucl Med.

[17] Gambhir SS, Schwaiger M, Huang SC, Krivokapich J, Schelbert HR, Nienaber CA (1989). Simple noninvasive quantification method for measuring myocardial glucose utilization in humans employing positron emission tomography and fluorine-18 deoxyglucose. J Nucl Med.

[18] Gheysens O, Postnov A, Deroose CM, Vandermeulen C, de Hoon J, Declercq R (2015). Quantification, variability, and reproducibility of basal skeletal muscle glucose uptake in healthy humans using 18F-FDG PET/CT. J Nucl Med.

[19] Thie JA, Hubner KF, Smith GT (2002). Optimizing imaging time for improved performance in oncology PET studies. Mol Imaging Biol.

[20] Lucignani G, Schmidt KC, Moresco RM, Striano G, Colombo F, Sokoloff L (1993). Measurement of regional cerebral glucose utilization with fluorine-18-FDG and PET in heterogeneous tissues: theoretical considerations and practical procedure. J Nucl Med.

[21] Vandenberghe S, Moskal P, Karp JS (2020). State of the art in total body PET. EJNMMI Phys.

[22] Surti S, Pantel AR, Karp JS (2020). Total Body PET: Why, how, what for?. Ieee T Radiat Plasma.

[23] Badawi RD, Shi H, Hu P, Chen S, Xu T, Price PM (2019). First human imaging studies with the EXPLORER total-body PET scanner. J Nucl Med.

[24] Zhang X, Cherry SR, Xie Z, Shi H, Badawi RD, Qi J (2020). Subsecond total-body imaging using ultrasensitive positron emission tomography. Proc Natl Acad Sci U S A.

[25] Pantel AR, Viswanath V, Daube-Witherspoon ME, Dubroff JG, Muehllehner G, Parma MJ (2020). PennPET Explorer: human imaging on a whole-body imager. J Nucl Med.

