# Supplementary material for: Assessment of population-based input functions for Patlak imaging of whole body dynamic 18F-FDG PET
Source: EJNMMI Phys. 2020 Nov 23;7:67. doi: 10.1186/s40658-020-00330-x (PMC7683759; doi:10.1186/s40658-020-00330-x)
Supplement: Supplementary file 1 — Additional file 1: Figure S1. (A) Three input functions simulated with different λ3 values (0%, 30%, 60% lower than mean value of 0.012 min-1) and the same area under the curve. Dotted curves show the difference from the input function with λ3=0.012; (B) two time-activity curves (TACs) computed using the input function (λ3=0.012). These curves have different Ki and the same Ve values, as specified in the legend; (C) Patlak plots of the TAC with the low Ki using the three input functions; (D) Patlak plots of the TAC with the high Ki using three input functions. Note the difference in y-axis scaling of (C) and (D). Table S1. Effect of λ3 of input function on the Ki estimation [file 40658_2020_330_MOESM1_ESM.docx]

**
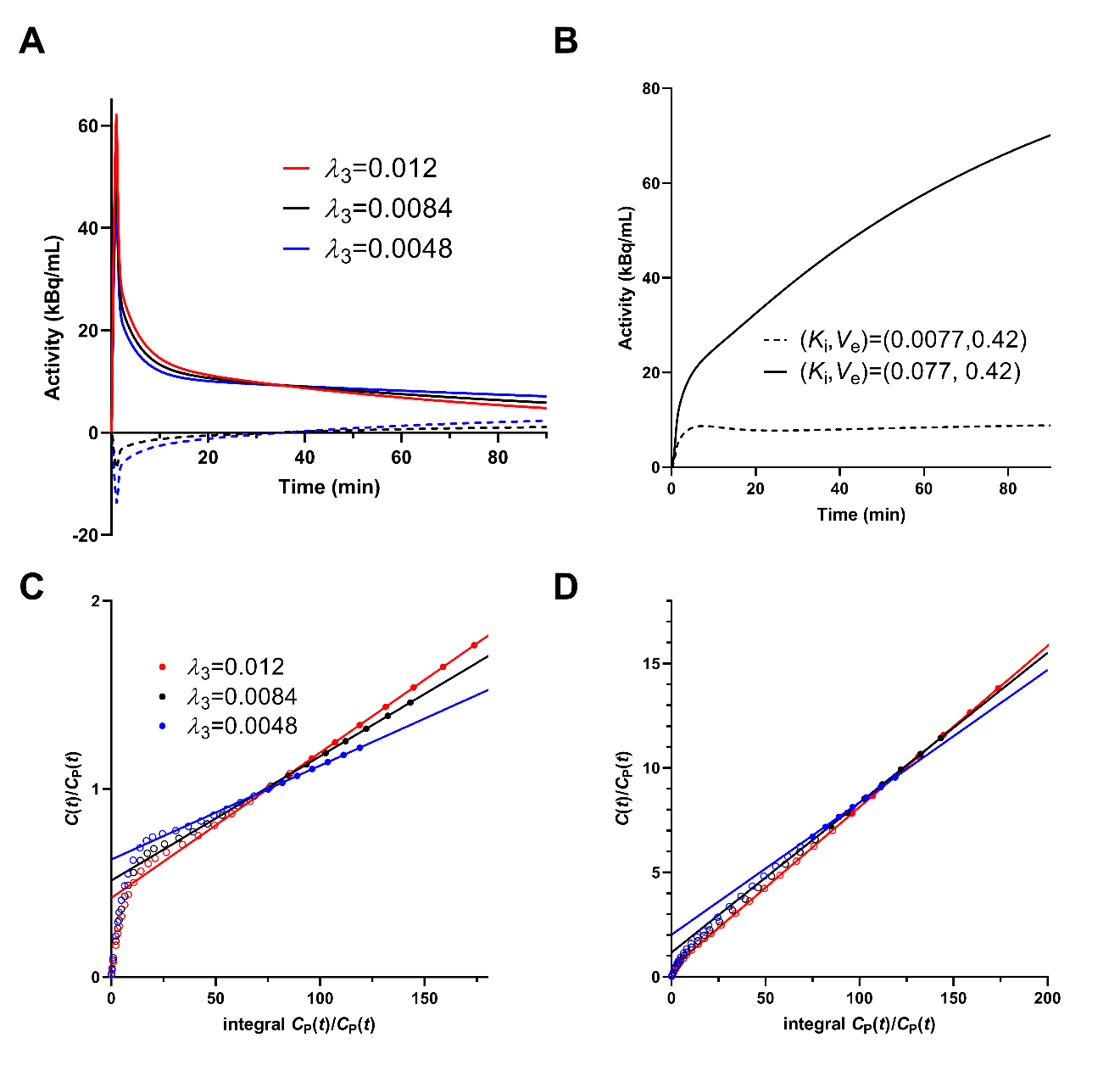
Figure S1: (A) Three input functions simulated with different** ***λ*_3_** values (0%, 30%, 60% lower than mean value of 0.012 min^-1^) and the same area under the curve. Dotted curves show the difference from the input function with ***λ*_3_**=0.012; **(B)** two time-activity curves (TACs) computed using the input function (***λ*_3_**=0.012). These curves have different *K*_i_ and the same *V*_e_ values, as specified in the legend; **(C)** Patlak plots of the TAC with the low *K*_i_ using the three input functions; **(D)** Patlak plots of the TAC with the high *K*_i_ using three input functions. Note the difference in *y*-axis scaling of **(C)** and **(D)**.

**Table S1: Effect of *λ*_3_ of input function on the *K*_i_ estimation**

| **Truth** | **Parameter** | ***λ*_3_** = 0.0084 (min^-1^) | ***λ*_3_** = 0.0048 (min^-1^) |
| --- | --- | --- | --- |
| **Low *K*_i_**  **(*K*_i_**, ***V*_e_)= (0.0077, 0.42)** | ***K*_i_** (mL/cm^3^/min) | **0.0066 (-14%)** | **0.0050 (-35%)** |
|  | ***V*_e_** (mL/cm^3^) | **0.51 (22%)** | **0.62 (49%)** |
| **High *K*_i_**  **(*K*_i_**, ***V*_e_)= (0.077, 0.42)** | ***K*_i_** (mL/cm^3^/min) | **0.071 (-7%)** | **0.063 (-18%)** |
|  | ***V*_e_** (mL/cm^3^) | **1.17 (177%)** | **2.02 (380%)** |
